# Exploring reasons and coping strategies among nursing students with nomophobia: a qualitative study

**DOI:** 10.3389/fpsyg.2026.1863185

**Published:** 2026-07-06

**Authors:** Jing Luo, Li Yoong Tang, Nor Aziyan Yahaya, Libo Zhou, Qiong Liu

**Affiliations:** 1 Department of Nursing Science, Faculty of Medicine, Universiti Malaya, Kuala Lumpur, Malaysia; 2 School of Nursing, Guizhou Medical University, Guiyang, Guizhou, China; 3 The Affiliated Hospital of Guizhou Medical University, Guiyang, Guizhou, China; 4 Guiyang Civil Aviation General Hospital, Guiyang, Guizhou, China

**Keywords:** nursing students, nomophobia, mental health, phone dependence, reason, coping, qualitative research

## Abstract

**Background:**

Accumulating research suggests that nomophobia is common among nursing students and has been linked to adverse outcomes.

**Objective:**

To explore the reasons for nomophobia and coping strategies employed by nursing students.

**Methods:**

This research employed a qualitative design. 13 s-year undergraduate nursing students in Guizhou Province, China with moderate to severe nomophobia were recruited using purposive sampling. Data were collected through semi-structured interviews and analyzed using thematic analysis. In the discussion section, the findings were interpreted through Maslow’s hierarchy of needs theory and Lazarus and Folkman’s transactional model of stress and coping.

**Results:**

Nursing students’ nomophobia was associated with perceived disruptions caused by smartphone unavailability in daily life functioning, academic routines, psychosocial support, and coping with developmental uncertainty. Additionally, coping strategies mainly involved sleeping or emptying the mind, diverting attention, and chatting with friends, while adopting alternative approaches was less frequently reported.

**Discussion:**

Nomophobia among nursing students may reflect the disruption of smartphone-supported resources related to multidimensional needs. Students appear to rely mainly on emotion-focused coping, which may provide only limited relief, whereas problem-focused coping is limited. Efforts to reduce nomophobia should consider educational support, mental health services, and individualized intervention design.

## Introduction

Smartphones have become deeply embedded in modern life across diverse groups (Bragazzi et al., 2019; Rodríguez-García et al., 2020). In 2025, approximately 5.28 billion people, about 65.4% of the global population, used smartphones (Turner, 2025). Global smartphone adoption is projected to reach 80% by 2029 (Turner, 2025). Nursing students need to devote much time and energy to in-depth learning of specialized knowledge and practical skills, and they increasingly rely on smartphones to complete academic tasks (Beauregard et al., 2017). Epstein and Bertram (2019) found that smartphone use supported reflective learning and skill development among nursing students. Moreover, in clinical practice, smartphone usage assists with various tasks such as accessing information quickly and communicating with peers and patients (O’Connor and Andrews, 2018). Previous research showed that 94.8% of nursing students carried their phones at all times, while 93.3% used them for study purposes, 89.6% participated in WhatsApp study groups, and 77.8% used them for in-class note-taking (Alsayed et al., 2020). These findings suggest that nursing students rely heavily on smartphones, leaving some of them more prone to nomophobia (Aguilera-Manrique et al., 2018; Çelik İnce, 2021; Çobanoğlu et al., 2021; Moreno-Guerrero et al., 2021; Uguz and Bacaksiz, 2022).

Nomophobia is the worry, anxiety, nervousness, or fear individuals experience when they cannot access or use their smartphones (Notara et al., 2021; Pavithra et al., 2015). It is particularly common among nursing students globally. A recent meta-analysis of samples from nine countries showed that 28% of nursing students had mild nomophobia, 49% had moderate nomophobia, and 15% had severe nomophobia (Mudgal et al., 2024). Surveying 933 undergraduate nursing students in China, Liu et al. (2024) found that 10.5% were mildly nomophobic, 53.6% had moderate nomophobia, and 35.9% had severe nomophobia. Such rates are alarming because nomophobia is accompanied by poor sleep quality and other sleep disturbances (Daraj et al., 2023; Jahrami et al., 2022; Ramjan et al., 2021). Furthermore, several psychological problems, including anxiety, depression, loneliness, and low self-esteem, are associated with nomophobia (Abdoli et al., 2023; Daraj et al., 2023; Gaber Hamzaa et al., 2024; Gutiérrez-Puertas et al., 2020; King et al., 2014; Santl et al., 2022; Servidio, 2023; Yigit et al., 2024). Besides, nomophobia may lead to poor academic motivation, low self-efficacy, and impaired attention and executive functioning among nursing students (Aguilera-Manrique et al., 2018; Gutiérrez-Puertas et al., 2020). Ultimately, these negative effects may hinder students’ learning ability and academic performance (Eskin Bacaksiz et al., 2022; Essel et al., 2021; Márquez-Hernández et al., 2020).

Although some studies have revealed the high prevalence and negative impacts of nomophobia, the bulk of research is quantitative, and few studies involve interventions designed for nursing students. Qualitative research can provide insights that inform contextually appropriate interventions (Yardley et al., 2021). However, current studies focus on other groups, such as university students (Ranjan et al., 2024; Safaria et al., 2025). As nursing education integrates not only academic study but also intensive skills training and clinical practice, nursing students often exhibit distinct psychological features compared with general university students (Bartlett et al., 2016). Thus, the results from studies on non-nursing students may not be directly applicable to nursing students with nomophobia. Moreover, the existing qualitative literature, with theoretical interpretations limited to attachment or self-extension perspectives, provides an insufficient explanation of the psychological mechanisms underlying nomophobia (Safaria et al., 2025). In addition, little is known about how nursing students cope with nomophobia. This gap results in insufficient understanding of coping strategies that could guide the development of feasible and student-centered interventions.

To fill in these gaps, we employed Maslow’s hierarchy of needs and Lazarus and Folkman’s transactional model of stress and coping to generate multiple perspectives to understand nomophobia among nursing students. According to Maslow’s (1987) theory, human behavior is motivated by a hierarchy of needs, which range from fundamental physiological requirements to higher-level self-actualization. Some studies suggest that smartphone use may meet individuals’ needs, including safety, belonging, and self-expression (Gezgin et al., 2018; Kang and Jung, 2014; Pavon, 2020). In this study, we used Maslow’s theory to illuminate the motivations for smartphone use that may contribute to nursing students’ experiences of nomophobia.

According to Lazarus (1993) transactional model of stress and coping, coping behaviors can be divided into problem-focused coping and emotion-focused coping. Zimmer-Gembeck (2021) reported that individuals who used a combination of problem-focused and emotion-focused coping strategies tended to cope with stress more effectively. In this study, we employed this model to provide a lens for discussing the coping strategies that nursing students used to manage nomophobia.

This study aimed to explore the reasons for nomophobia and the coping strategies adopted by nursing students. It further sought to provide in-depth, nursing-specific qualitative evidence for developing future interventions. To achieve this aim, the study was guided by the following research questions:

(1) Why do nursing students experience nomophobia?
(2) How do nursing students cope with nomophobia?

## Methods

### Design

This study employed a qualitative descriptive design to explore individuals’ experiences and to generate common themes from the data (Willis et al., 2016). In qualitative descriptive research, researchers conduct a certain level of interpretation based on results to identify common patterns and facilitate understanding (Vaismoradi et al., 2013). In this study, the qualitative descriptive design was selected over other qualitative approaches because it supports the generation of practice-relevant knowledge to inform intervention development, rather than the development of theory or the exploration of the philosophical essence of experience (Willis et al., 2016). In the discussion section, the findings were interpreted through the theoretical lenses of Maslow’s hierarchy of needs and Lazarus and Folkman’s transactional model of stress and coping.

### Participants

We recruited second-year undergraduate nursing students from a university in Guizhou Province, China. Second-year students were selected based on the research team’s observations. At this stage, students appeared to be undertaking more independent academic responsibilities and were observed to use smartphones frequently for learning and daily communication, while their professional identity and coping strategies were still developing. At the beginning of recruitment, counselors introduced the study aims to students and invited those who were interested to participate. Interested students then completed an online questionnaire, the Chinese version of the Nomophobia Questionnaire (NMP-C), revised by Ren et al. (2020), for preliminary nomophobia screening. The NMP-C consists of 16 items across four dimensions: fear of being unable to obtain information, losing convenience, losing contact, and losing internet connection. Each item is rated on a 7-point Likert scale, with nomophobia scores ranging from 16 to 112. Based on the classification proposed by the original scale developers (Yildirim and Correia, 2015), scores were categorized as mild (17–48), moderate (49–80), and severe (81–112). The Cronbach’s alpha coefficient for the overall NMP-C was 0.931 (Ren et al., 2020). After the students voluntarily completed the electronic questionnaire, we identified 50 nursing students with moderate or severe nomophobia who were willing to participate in the study. This study focused on these students to obtain rich and in-depth data. From this eligible pool, we adopted purposive sampling to select participants. In this study, we had no pre-existing relationships with the participants. Besides, we did not teach or grade the participants.

The inclusion criteria were as follows: (a) being full-time second-year undergraduate nursing students; (b) owning a smartphone; (c) having moderate or severe nomophobia, defined as an NMP-C score >48; and (d) being willing to participate and providing written informed consent. The exclusion criteria were as follows: (a) being currently on leave of absence, under suspension, or with irregular enrollment status; (b) presence of acute psychological crisis or severe psychiatric symptoms, or recent major life events within the past three months that could significantly affect psychological status; and (c) inability to complete the interview due to cognitive, language, hearing, or other communication impairment.

### Data collection

We collected data between October and November 2025. Semi-structured interviews were used to collect data. The interview guide was developed based on the purpose of this study and the relevant literature (Yousefian and Khodabakhshi-Koolaee, 2023). It was refined through expert consultation and preliminary interviews. The formal interview guide included: “please describe recent situations when you could not use your phone,” “how did you feel when your phone was not with you?,” “what do you think contributes to these feelings when you are unable to use your phone?,” and “what did you do to cope with those feelings?”

Interviews were conducted either face to face or via video calls. The face-to-face interviews were held at locations selected by the participants, such as the college counseling room or a seminar room, all of which provided a sufficiently quiet and private environment. Interviews lasted between 40 and 60 min. All interviews were audio-recorded. Based on insights gained from earlier interviews, the interviewer adjusted subsequent questions to better understand participants’ nomophobia and their coping strategies. Brief field notes were taken during each interview. Recordings were transcribed verbatim shortly after the interview. Audio files and transcripts were anonymized and stored on password-protected devices accessible only to the research team. Two researchers independently checked each transcript against the recording. Any discrepancies were discussed with the interviewer and resolved through joint review. Besides, transcripts were returned to participants within 48 h for confirmation. All participants confirmed their transcripts within the 48-h timeframe. Thus, no follow-up procedures for delayed responses were required. No participant requested substantial changes. Thematic saturation was first indicated at the 11th interview, when no new themes emerged. Two further interviews were conducted to confirm saturation, resulting in a total of 13 interviews with 13 participants. Each participant completed a single interview, and none withdrew from the study after enrollment.

### Data analysis

Thematic analysis was employed (Braun and Clarke, 2006). The analysis was conducted inductively. First, the researchers repeatedly read the verbatim transcripts together with the field notes to become familiar with the data. Meaningful data segments relevant to the research questions were then identified, and initial codes were generated from participants’ accounts rather than from pre-established theoretical categories. Through ongoing comparison, categorization, and integration of related codes, preliminary subthemes and themes were developed.

Throughout the analysis, two researchers worked independently (one with a nursing-education background and one with a background in psychology). All research team members met to discuss the emerging codes, subthemes, and themes until consensus was reached. To enhance interpretive depth, participants were re-engaged after the preliminary analysis to provide participatory feedback to question, clarify, and add nuance to interpretations (Maher, 2025). Of the 13 participants, 11 participated in the participatory feedback stage following the preliminary analysis. The remaining two participants did not respond to the invitation to provide feedback. No revisions to the preliminary findings were requested by the participants who provided feedback.

### Trustworthiness

To ensure that the study’s interpretations were grounded in participants’ experiences, the research team implemented reflexive practices throughout the data collection and analysis process. In particular, the interviewer and analysts repeatedly reflected on how their professional roles and preconceptions might shape the interview process, coding decisions, and theme development, including potential influences related to teacher–student power dynamics, professional terminology, and educational or psychological interpretations of students’ mobile phone use. To enhance analytic transparency, the interviewer and analysts maintained reflective journals, while other team members contributed reflexive memos, methodological oversight, and critical discussion during team meetings.

The study followed Lincoln and Guba’s (1986, 1988) four trustworthiness criteria: credibility, dependability, confirmability, and transferability. To enhance credibility, all interview transcripts were cross-checked and any discrepancies were resolved through discussion with the interviewer. In addition, participants reviewed the transcripts to ensure accuracy. Preliminary interpretations were further validated through follow-up member checking with participants. Dependability was strengthened through an audit of the analytic process conducted by an external qualitative researcher, using verbatim transcripts, field notes, records of coding revisions, and reflexive journals. To support confirmability, analyst triangulation was also employed, involving two researchers from different professional backgrounds. Besides, analysts kept reflexive journals to document assumptions and track analytic decisions, thereby minimizing potential researcher bias. Finally, to support transferability, detailed descriptions of the research context, participant characteristics, inclusion criteria and their rationale, as well as data collection and analysis procedures were provided to allow other researchers to assess the applicability of the findings to similar contexts.

### Ethical considerations

This study was approved by the Ethics Committee of Guizhou Medical University (Approval Number: 2025 Lunshen No. 343). It followed the Declaration of Helsinki, with full respect for participants’ privacy and careful handling of all data. Before the study began, participants were given a clear explanation of its purpose in plain language. They were also informed that participation was voluntary and that their information would remain confidential. All participants signed written informed consent forms before the interviews.

During the interviews, if participants showed any signs of physical or psychological discomfort, the interviewer would respond immediately to ease the situation. If the discomfort continued, the interview would be paused and rescheduled. No participants experienced serious discomfort during the study.

## Result

### The characteristics of students

The characteristics of the nursing students were presented in Table 1. The students’ mean age was 19.92 years (standard deviation [SD] = 1.12), ranging from 19 to 23 years. Among these students, four (31%) were male and nine (69%) were female. These demographic characteristics show that the second-year participants are young and predominantly female nursing students. The cumulative grade point average (GPA) was 2.93 (SD = 0.22), with values ranging from 2.1 to 3.3. According to the university grading system, participants’ cumulative GPA scores were distributed within a limited range, suggesting relatively similar academic performance across the sample. Regarding smartphone usage, 53.8% of students reported using smartphones for 5–10 h per day, 23.1% for 2–4 h, and 23.1% for more than 10 h per day. These findings indicate that smartphone use is common among the participants, with most reporting smartphone use for five hours or more each day. The mean score on the NMP-C was 77.46 (SD = 15.46), with scores ranging from 49 to 106. Based on the scoring criteria, the average NMP-C score indicates a moderate level of nomophobia, while the observed score range shows that participants’ nomophobia levels vary from moderate to severe.

**Table 1:** The characteristics of the nursing students.

Characteristics (*N* = 13)	Category/value	*N* (%)
Age (years)	19.92 ± 1.12 (min:19; max: 23)	-
Gender	Male	4 (31%)
	Female	9 (69%)
Cumulative GPA (5.0-point scale)	2.93 ± 0.22 (min: 2.1; max: 3.3)	-
Daily mobile phone usage (hours/day)	2–4	3 (23.1%)
	5–10	7 (53.8%)
	>10	3 (23.1%)
NMP-C scores	77.46 ± 15.46 (min:49; max:106)	-

Values are presented as mean ± standard deviation for continuous variables and n (%) for categorical variables. GPA, grade point average, cumulative GPA was calculated on a 5.0-point scale according to the university grading system, where percentage scores below 60 corresponded to 0, 60–69 to 1.0–1.9, 70–79 to 2.0–2.9, 80–89 to 3.0–3.9, 90–99 to 4.0–4.9, and 100 to 5.0; NMP-C, Chinese version of the nomophobia questionnaire. 0 participants reported <2 h.

### Themes

This study was structured around two research focuses: “reasons for nomophobia” and “coping strategies.”

As shown in Table 2, four themes and 10 subthemes were identified in relation to the reasons for nomophobia: disruption of daily life functioning, disruption of academic routines, perceived loss of psychosocial support, and disruptions to coping with developmental uncertainty. These themes suggest that nursing students’ nomophobia is related to smartphone unavailability that disrupts aspects of their daily routines, learning, social relationships, emotional regulation, and personal development.

**Table 2:** Themes and subthemes identified under the two research focuses: “reasons for nomophobia” and “coping strategies.”

Research focus	Themes	Subthemes
Reasons	Disruption of daily life functioning	Inability to make digital payments
		Perceived loss of navigation support
		Disruption of routine-sustaining information exchange
		Inability to access immediate emergency assistance in safety-threatening situations
	Disruption of academic routines	—
	Perceived loss of psychosocial support	Perceived loss of social connection
		Perceived loss of social coping resource
		Perceived loss of self-acceptance resource
	Disruptions to coping with developmental uncertainty	Lack of purposeful activities during free time
		Perceived loss of academic and career guidance
		Perceived loss of access to volunteering
Coping strategies	Sleeping or emptying the mind	—
	Diverting attention‌	—
	Chatting with friends	—
	Adopting alternative approaches	—

The theme of disruption of daily life functioning included four subthemes: inability to make digital payments, perceived loss of navigation support, disruption of routine-sustaining information exchange, and inability to access immediate emergency assistance in safety-threatening situations. These subthemes indicate that participants rely on smartphones to manage essential daily activities. The theme of disruption of academic routines was identified as an independent theme without further subthemes, suggesting that participants also associate smartphone access with learning-related routines. The theme of perceived loss of psychosocial support included three subthemes: perceived loss of social connection, perceived loss of social coping resource, and perceived loss of self-acceptance resource. These subthemes show that participants view smartphones as a means of maintaining social contact and emotional support. The theme of disruptions to coping with developmental uncertainty included three subthemes: lack of purposeful activities during free time, perceived loss of academic and career guidance, and perceived loss of access to volunteering. These findings suggest that participants also link smartphone use to development-related personal goals, relevant guidance, and opportunities.

Four coping strategy themes were identified: sleeping or emptying the mind, diverting attention, chatting with friends, and adopting alternative approaches. In contrast to the themes related to the reasons for nomophobia, these results suggest that participants’ coping strategies are described at a broader thematic level and are not further organized into subthemes.

### Reasons for nomophobia

#### Theme 1: Disruption of daily life functioning

This theme described how nursing students experienced nomophobia when smartphone unavailability disrupted their daily life functioning. In this theme, daily life functioning refers to the activities and resources students rely on to manage essential everyday tasks, such as making digital payments, maintaining wayfinding ability during daily mobility, exchanging information needed to maintain daily routines, and obtaining immediate assistance in emergencies. When access to these smartphone-supported functions was interrupted, students felt less able to maintain their daily functioning. As a result, they experienced unease, anxiety, and fear, which contributed to their experience of nomophobia.

##### Subtheme 1: inability to make digital payments

In highly digitalized contexts, the tendency to go cashless was linked to nomophobia, as digital payments via smartphones had become the primary payment method for transportation. As one student reported:

*“I rarely carry cash since I use Alipay for everything. If my phone dies while I’m out, I get really anxious, because I might have no way to pay my fare to get back to school. And it’s not like the old days, it’s so hard to borrow a phone or cash now, and public phones are basically extinct…”* (P5).

##### Subtheme 2: perceived loss of navigation support

For a few nursing students, smartphone navigation systems supported their sense of safety to some extent and reduced their concerns about potential personal safety risks. Once their phones were unavailable, they gave way to uneasy feelings and heightened perceptions of risk. As one student described:

*“I’m terrible with directions, and thus I rely heavily on GPS. Without my phone, I’d totally freak out, and I’d be afraid of getting lost or even stranded in a risky area.”* (P1).

##### Subtheme 3: disruption of routine-sustaining information exchange

The inability to engage in routine-sustaining information exchange with teachers, classmates, family members, and work-related contacts was another contributing factor to nomophobia. Most nursing students frequently mentioned the fear of missing messages regarding urgent administrative meetings, non-academic student affairs, and family-related matters. As one student reported:

*“If I miss important class announcements, I get scared…because it feels like I’ve lost control over things I’m supposed to know.”* (P3).

One student leader indicated that phone separation caused feelings of anxiety, as they were afraid of missing key information and failing to fulfill their duties on time:

*“If I do not have my phone, I will not be able to receive messages and may miss something important…When teachers need me to contact my classmates (as the class monitor) and I cannot do it on time, I might even be held responsible, which makes me feel very anxious…”* (P4).

Besides, another student stated that their delivery job depended entirely on the phone to receive orders and contact customers. Without the phone, they could not complete their work tasks, which reduced their earnings and fueled their anxiety.

##### Subtheme 4: inability to access immediate emergency assistance in safety-threatening situations

In safety-threatening situations, being unable to contact others for immediate help through the smartphone contributed to nomophobia. A few nursing students regarded the smartphone as an essential tool for seeking assistance when they felt unsafe. One nursing student described “clutching the phone” in preparation for calling the police and stated:

“*If I do not have it, I feel really scared. It feels like even if I screamed for help, no one would hear me*.” (P5).

Besides, situations in which students were alone and perceived threats to their personal safety were another factor contributing to nomophobia. For instance, one student experienced nomophobia after hearing strange noises while living alone at night, as the absence of a phone made it difficult to seek help immediately. Another student noted:

“*When I take a taxi, if something happens and I am in danger, I use my phone to call others. Without it, I would feel uneasy and nervous.*” (P11).

#### Theme 2: Disruption of academic routines

Smartphones had become deeply embedded in nursing students’ academic routines, and their convenience and accessibility were closely linked to nomophobia. Most nursing students mentioned that they were accustomed to using smartphones to access course materials, complete study tasks, watch instructional videos, and receive learning-related notifications anytime and anywhere. When smartphones were unavailable, they could not complete academic activities smoothly, leading to anxiety. One nursing student highlighted the indispensable role their phone played in their studies. Phone separation would lower their study efficiency, triggering anxiety.

*“…For our assignments, the textbook alone is not enough to get this done. We cannot complete them well. Having a phone lets us look things up online and add to what we know, which helps us do a better job. Besides, textbooks are usually so thick and heavy to carry around, whereas a phone is much more convenient. I can save everything on it, like taking photos of the pages to read directly on my screen. It’s been so helpful for my study that I have become dependent on it; I feel like I need it for everything now. If I do not have it with me, I feel really anxious.”* (P9).

Besides, some digital course designs shaped the experience of nursing students with nomophobia. Students were required to use their smartphones to complete classroom activities‌, such as attendance recordings and in-class quizzes. As one student noted:

*“Because we need to scan quick response codes to enter Rain Classroom (a mobile-based learning platform) to record attendance and complete quizzes, I feel uneasy without my phone.”* (P12).

#### Theme 3: Perceived loss of psychosocial support

In this theme, psychosocial support refers to the social and emotional support that students perceived as being mediated through smartphone use. Smartphones helped students maintain social connection, buffer social discomfort, and sustain self-acceptance. When students lost access to their smartphones, their anxiety stemmed from the sudden severance of the social connection, comfort, and self-acceptance on which they depended through their smartphones.

##### Subtheme 1: perceived loss of social connection

Nursing students described smartphones as essential tools for maintaining close relationships and alleviating loneliness. When they were unable to use their phones, they experienced anxiety not only because relationship maintenance was disrupted, but also because they felt lonely. For some students, smartphones were crucial in intimate relationships and close friendships, helping them respond to important others in a timely manner and manage interpersonal tensions.

*“…my girlfriend. If I do not reply to her messages in time, she gets angry and thinks I do not care about her. That really makes me anxious…”* (P4).

*“If my friend is emotionally unstable and I do not have my phone to reach him, I’d feel very anxious…I’d worry he might do something extreme.”* (P7).

*“When I have conflicts with my close friends, I usually use my phone to contact others and figure out the problem. If I do not have my phone with me, I feel really anxious, because I do not know how to handle it on my own…”* (P11).

A few students emphasized that smartphones enabled them to stay in touch with family and friends who lived far away, and that smartphone unavailability created a sense of loneliness.

*“…Living alone in another city…if I have no phone, I cannot contact my family, and I feel extremely anxious.”* (P11).

*“…Other things can temporarily ease loneliness…but when I feel very lonely, I really need someone to talk to. When I do not have my phone, I get super anxious… Like, seriously, I feel like… I do not know, like I cannot reach anyone, like I’m just completely cut off from the world.”* (P12).

One student reported that the phone provided them with psychological comfort, while phone separation made them restless.

*“My phone is like a companion… even when I’m not really talking to friends or family, just looking at the screen and checking if someone has messaged me gives me reassurance. But when I do not have it, I start to become restless, like something important is missing…”* (P5).

##### Subtheme 2: perceived loss of social coping resource

Nomophobia was associated with a perceived loss of social coping resource among nursing students, particularly in unfamiliar interpersonal situations. Most students shared that when communicating with strangers, they felt awkward and worried about saying something inappropriate. To alleviate this discomfort, smartphones were often used to avoid direct interaction and fill conversational gaps. When smartphone use was impossible, they found it more difficult to handle these situations, triggering feelings of unease and anxiety. As one student noted:

*“When I talk with others, I feel nervous…it feels like every word I say just comes out without thinking…Maybe I’m not good at expressing myself. In this situation, I’m afraid to talk to strangers. I avoid embarrassment and immerse myself in my own world through my phone…Without it, I would feel really uneasy…like I would not know what to do with myself.”* (P6).

##### Subtheme 3: perceived loss of self-acceptance resource

One nursing student described using their smartphone as a source of self-encouragement when struggling with aspects of themselves that they found it difficult to accept. Reading inspirational quotes on the phone appeared to help them counter negative self-perceptions and regain a sense of reassurance. When the phone was unavailable, this source of encouragement was disrupted, making self-critical thoughts difficult to manage and intensifying anxiety.

*“In some respects, I find it hard to accept myself…Sometimes I read inspirational quotes on my phone to encourage myself…But when I do not have my phone with me, I lose that source of encouragement fuels my anxiety…I’m not doing anything right…”* (P12).

#### Theme 4: Disruptions to coping with developmental uncertainty

Personal development involves setting personal goals, obtaining relevant guidance, and accessing opportunities that support individual growth (Ryan and Deci, 2000). In this study, developmental uncertainty referred to students’ uncertainty about what they should do, how to obtain academic- and career-related information, and how to access opportunities related to their growth, such as volunteering. This theme described how smartphone separation was perceived by students as disrupting their ability to cope with such developmental uncertainty. For some students, smartphones helped them manage their free time, seek academic and career-related information, and access volunteering opportunities. When smartphones were unavailable, these coping resources were disrupted, making students more aware of their lack of clear goals, insufficient guidance, or limited access to volunteering opportunities. This perception further contributed to nomophobia.

##### Subtheme 1: lack of purposeful activities during free time

To achieve self-development, individuals need clear personal goals that guide them toward meaningful and purposeful action (Emmons, 2003). In this study, some nursing students described uncertainty about how to use their free time. Smartphones appeared to help them manage this uncertainty by providing a sense of occupation. When phones were unavailable, students became more aware of having “nothing to do,” which was accompanied by anxiety. As one student stated:

*“I rely on my phone because I cannot find anything to do…having a phone makes me feel like I’m doing something…I’m not really sure what I’m supposed to be doing most of the time. But without my phone, I start to feel anxious like I cannot fix it…”* (P1).

In addition, another student suggested that the less structured nature of university life made the absence of a smartphone more difficult to tolerate.

*“In high school I was very busy all the time. I did not even care much about not having a phone. But now? There’s not much to do…When I do not have my phone, time just slows down to a crawl… I start to feel anxious. I honestly cannot imagine life without it.”* (P13).

These accounts suggest that, for some students, separating from smartphones was linked to a lack of purposeful activities to spend free time. Without smartphones, this lack of direction became more salient, intensifying feelings of emptiness, slowed time, and nomophobia.

##### Subtheme 2: perceived loss of academic and career guidance

Some nursing students relied on smartphones to get academic and career development guidance. These students reported feeling anxious when they were unable to use smartphones. Students leveraged the search and communication functions of their phones to access information related to personal growth. One student searched for relevant guidance online, stating:

*“Nowadays, I can find almost everything on the internet. The search function on my phone makes it easy for me to seek help, such as advice on how to do better in my study, recommendations for good courses, and guidance related to career development in my major…Without it, I’m stuck, like I’ve lost my guide. This makes me feel anxious.”* (P4).

Another nursing student described seeking related advice through smartphone communication:

*“I have no idea about my future plans. I do not know who to ask. Sometimes I want to text senior students or alumni to seek advice. Without the phone, I would feel very anxious…”* (P12).

These accounts show that smartphones are perceived as developmental support tools through which students’ access academic- and career-related information. When smartphones are unavailable, students feel that the important source of guidance has been removed, which intensifies their uncertainty about academic and career development.

##### Subtheme 3: perceived loss of access to volunteering

One student indicated that their phone helped to compensate for their limited communication skills. Without the phone, they had no way to learn about volunteer activities, which contributed to nomophobia.

*“I use my phone for almost everything, like finding and signing up for volunteer activities. If I do not have my phone, I will not even hear about that stuff. Because my communication skills are poor, I might not even dare to ask people.”* (P5).

This finding suggests that, for students who perceive themselves as having limited communication skills, smartphones may serve as an important bridge to accessing volunteering opportunities, and smartphone unavailability can contribute to nomophobia.

### Coping strategies‌

#### Theme 1: Sleeping or emptying the mind

Some students noted that they coped with nomophobia by sleeping or emptying their minds. These strategies functioned as a form of temporary emotional disengagement. For some, sleep was a simple way to stop overthinking and disconnect from anxious thoughts. One student reported that sleep was helpful in reducing mental fatigue and anxiety:

*“When I sleep, I do not think too much. It helps my mind and body rest, so I do not feel anxious.”* (P5).

Similarly, emptying the mind allowed students to suspend anxious thoughts and regain a sense of calm. One participant described this strategy as a deliberate effort to narrow attention and stop intrusive thinking:

*“I usually try to look at one spot and not think about anything. It usually works…”* (P6).

However, these strategies appeared to provide only temporary relief and were less helpful when students had nothing else to do. One participant noted:

*“When there’s nothing else to do, sleeping does not really help with anxiety.”* (P8).

#### Theme 2: Diverting attention‌

Most students coped with nomophobia by engaging in activities they enjoyed. Such activities distracted them from anxiety. Exercise was a prominent distraction strategy, mentioned by seven of the 13 students. Exercise-related activities included yoga, badminton, basketball, table tennis, jumping rope, running, and workouts guided by fitness videos. One student described how exercise helped them focus on the activity rather than on their anxiety:

*“I fully immerse myself in the exercise…like when I play badminton, I focus on how to hit the shuttlecock without sending it out of bounds or into the net. And then I’m no longer anxious.”* (P2).

In addition to exercise, some students alleviated their anxiety through leisure activities, including listening to music, drawing, doing makeup, reading, playing video games, singing, watching movies, and playing mahjong. These activities helped them relax and shift their attention toward more engaging, real-world experiences.

A few students noted that the effectiveness of these activities, particularly listening to music, depended on the severity of their nomophobia. In mild cases, music could be helpful, but it might be ineffective for students experiencing more severe nomophobia.

#### Theme 3: Chatting with friends

Chatting with friends was another strategy used by students to cope with nomophobia. Through conversations with peers, students were able to receive comfort and reduce the emotional burden caused by phone separation. This strategy was especially helpful when nomophobia was related to missing important messages or interpersonal concerns. One participant described seeking comfort from friends when they were worried about missing important information from a teacher:

*“I complain to my friends if I do not receive my teacher’s message and fail to complete a task on time. They comfort me, and I do not feel anxious anymore.”* (P4).

Another participant described talking with other friends as a way to relieve negative emotions when they experienced interpersonal conflict but did not have their phone with them:

*“I usually talk with my friends. The anxiety fades away after chatting for a while. It helps me let go of negative emotions.”* (P11).

#### Theme 4: Adopting alternative approaches

A few students noted that when they were without their phones, they would actively cope with nomophobia through alternative means, such as using an iPad, borrowing someone’s phone, or seeking other solutions. As one student reported:

*“(When I do not have my phone.) I look for substitutes, like an iPad or a computer If I need to contact someone, I find another phone.”* (P7).

Unlike the previous strategies, this theme reflected students’ attempts to replace some of the functions usually provided by smartphones, including communication, access to information, and task completion. However, such strategies were reported by only a few participants and were mainly limited to functional substitution, suggesting that students described relatively few practical strategies for directly dealing with nomophobia.

## Discussion

This study explored why nursing students developed nomophobia and what coping strategies they used. The findings indicated that students’ nomophobia was linked to their functional and psychological reliance on smartphones across four domains: daily life functioning, academic routines, psychosocial support, and coping with developmental uncertainty. Maslow’s hierarchy of needs was used as an interpretive lens to explain how smartphone unavailability might be perceived as threatening students’ needs for safety, belongingness, esteem, and self-actualization. In addition, the findings showed that nursing students employed several coping strategies to manage nomophobia, including sleeping or emptying the mind, diverting attention, chatting with friends, and adopting alternative approaches. Lazarus and Folkman’s transactional model of stress and coping was used as an interpretive lens to understand these strategies in terms of emotion-focused coping and problem-focused coping.

This study found that nursing students’ nomophobia was closely tied to their reliance on smartphones as a means of sustaining the functioning of daily life. Smartphone-supported functions, including digital payments, navigation, routine information exchange, and emergency help-seeking, enabled nursing students to manage everyday routines, maintain a sense of predictability in daily life, and respond to unexpected situations. When smartphones were unavailable, students perceived threats to control over daily life and personal safety. From the perspective of Maslow’s hierarchy of needs, such experiences can be interpreted as reflecting a perceived threat to safety needs, which encompass the need for security, stability, and control over one’s environment (Maslow, 1943). Notably, although some smartphone-supported functions, such as navigation, may be closer to basic daily or physiological needs because they support routine mobility and access to everyday resources, participants in this study described these functions mainly in safety-related terms (e.g., “…I’d be afraid of getting lost or even stranded in a risky area…” P1). Therefore, these functions were perceived as being related to safety needs. Empirical research suggested that safety needs showed the strongest association with anxiety among Maslow’s need domains (Saunders et al., 1998). Thus, nomophobia triggered by smartphone unavailability may reflect students’ safety-related concerns in situations where smartphones were perceived as needed to maintain security, stability, and control in daily life.

In this study, smartphones were perceived as an important source of psychosocial support for nursing students, helping them maintain social connections, buffer social discomfort, and sustain self-acceptance. Some nursing students used smartphones to maintain social connections, and the inability to use smartphones could trigger anxiety. This is consistent with prior research suggesting that nomophobia may be associated with fear of losing social connectedness (Farooq et al., 2022). From the perspective of Maslow’s hierarchy of needs, this finding may reflect students’ love and belonging needs, as smartphones help them maintain interpersonal ties and reduce feelings of social disconnection. In addition to maintaining social connectedness, nursing students’ experiences of nomophobia appeared to be associated with a lack of social coping resource and self-acceptance resource. For example, most students noted that when communicating with strangers, they were worried about saying something inappropriate and often used phones to avoid direct interaction. One nursing student further suggested that smartphone use could provide a sense of self-encouragement and self-acceptance. These findings can be interpreted within the “esteem needs” domain of Maslow’s hierarchy. Consistent with this interpretation, prior studies suggested that individuals with low self-esteem were more likely to use smartphones to avoid real-life social interactions (Edwards et al., 2022; Putri and Wibowo, 2024), and negative self-evaluations may further increase the risk of nomophobia (Gonçalves et al., 2020). Thus, these findings provide additional insight into how smartphones may support nursing students’ psychosocial needs by maintaining social connectedness, reducing social discomfort, and offering a sense of self-encouragement in daily life.

In this study, students’ nomophobia was linked to a perceived disruption to coping with developmental uncertainty when smartphones were unavailable. This disruption was reflected in their perceived lack of purposeful activities to occupy free time, perceived loss of academic and career guidance, and perceived loss of access to volunteering opportunities. From the perspective of Maslow’s hierarchy of needs, these experiences may reflect concerns related to self-actualization, which involves the continual pursuit of personal growth, the realization of one’s potential, and the completion of unfinished goals (Maslow, 1962). Sub-themes such as perceived loss of academic and career guidance (e.g., “I do not know who to ask”; P12), and perceived loss of access to volunteering (e.g., “I will not even hear about that stuff”; P5) suggest that nursing students are in an exploratory developmental stage and may have limited support in navigating academic, career, and extracurricular opportunities. These findings are consistent with previous evidence showing that guidance and support for Chinese nursing students in academic and career development remain insufficient (Chen et al., 2023; Wang et al., 2024). This study further suggests that, when such support was perceived as insufficient, nursing students may have turned to smartphones to seek academic and career information and personal development opportunities, which may have contributed to their nomophobia. Besides, in this study, some students used smartphones as a compensatory resource to occupy their free time and gain a sense of “doing something” during free time. Without their phones, they felt that they had no personal goals and nothing to do, which made them anxious. This finding is similar to the findings of Zhang et al. (2023), which showed that boredom proneness mediated the relationship between anxiety and smartphone addiction. One student mentioned that having more free time in university made it harder for them to stay off their phone. This finding supports previous evidence, which suggests that having more free time without enough guidance may lead to negative behavioral outcomes (Patzak and Zhang, 2025).

This study found that nursing students relied heavily on smartphones in their learning activities to access course materials, complete assignments, and participate in classroom interactions. When smartphones were unavailable, learning activities were hindered, and students might experience increased nomophobia, which can be interpreted as reflecting the unmet satisfaction of their cognitive needs. Cognitive needs involve an individual’s desire for knowledge, understanding, and exploration, and constitute an important component of self-actualization needs (Maslow, 1987). This finding suggests that smartphones become an important medium through which nursing students satisfy their cognitive needs and support their professional growth and self-actualization. Notably, this study found that digital course design, such as attendance recordings, might be associated with nomophobia. This suggests that course design requiring frequent smartphone use may inadvertently exacerbate nomophobia (Gözümi et al., 2020). Previous scholars suggested encouraging the academic use of digital devices while limiting non-academic and potentially distracting uses during class time (Rogayan and Abaniel, 2025). Thus, educators may consider avoiding unnecessary smartphone-based course designs, which may help reduce students’ situational dependence on smartphones.

Overall, our findings suggest that nomophobia among nursing students may be understood as more than discomfort caused by the absence of a technological device. Instead, nursing students’ accounts indicate that smartphone unavailability is experienced as a disruption to resources that help them maintain daily life functioning and academic routines, obtain psychosocial support, and engage in developmental exploration. From the perspective of Maslow’s hierarchy of needs, these disrupted functions reflect students’ needs for safety, belongingness, esteem, and self-actualization, which smartphones appear to support as multifunctional resources. When smartphones become unavailable, these need-supporting resources are perceived as temporarily inaccessible, leaving related needs insufficiently supported. Previous research showed that lower satisfaction of basic needs was associated with anxiety, depression, and anger (Saunders et al., 1998). Thus, in this study, nomophobia may be interpreted as a perceived threat to need-supporting resources rather than simply as dependence on a device.

In terms of coping strategies, we found that when facing nomophobia, most nursing students tended to use strategies, such as sleeping or emptying the mind, diverting attention, and chatting with friends. Following Lazarus and Folkman’s (1984) theoretical framework, these strategies are categorized as emotion-focused because they primarily regulate the emotional response to stress rather than addressing the issue itself. This finding aligns with previous studies reporting that students prefer emotion-focused coping (Bista et al., 2017; Fornés-Vives et al., 2016; Nway et al., 2023). Besides, previous researchers have shown that emotion-focused coping can temporarily alleviate stress (Hirsch et al., 2015). Our findings further support this finding, suggesting that exercise or listening to music helps to alleviate nomophobia but does not directly mitigate the functional limitations caused by being without a mobile phone.

According to Lazarus and Folkman (1984), problem-focused coping strategies seek to resolve the source of issue rather than merely reduce the emotional impact. However, in our study, nursing students rarely reported such strategies beyond functional substitution strategies (e.g., using a computer or paper notes as temporary alternatives to a smartphone), suggesting a limited range of problem-focused coping strategies when confronting nomophobia. Thus, future nomophobia interventions should incorporate problem-focused coping training.

Another important finding was that students used attention-diversion strategies to cope with nomophobia. This finding resonates with research by Anjana et al. (2021), which found that distraction was commonly used by medical students to manage nomophobia. But our study further indicates that exercise is a frequently used strategy among nursing students. In this study, participants also tended to select attention-diversion strategies based on personal interests, indicating that intrinsic motivation plays a central role in coping behavior. As Renninger and Hidi (2015) noted, interest was a core component of intrinsic motivation that fostered engagement and persistence. In line with this, Self-Determination Theory suggests that activities driven by intrinsic motivation are more likely to be sustained because they are inherently enjoyable and self-endorsed (Ryan and Deci, 2000). This finding suggests that future interventions may consider students’ personal interests.

### Implications

Our findings suggest that nursing schools and educators may consider incorporating needs-based assessments into academic and clinical settings to identify unmet needs potentially associated with nomophobia and to inform individualized support. Besides, interventions tailored to students’ personal interests may enhance their engagement.

In educational practice, nursing curricula and student development programs may support students’ academic and career development by providing academic guidance, career planning, and accessible information related to academic and career development, which may help to reduce students’ reliance on smartphones as a primary source of support. For learning activities where smartphone use is not essential, educators may provide alternative participation options, such as printed materials or offline resources, to reduce situational dependence on smartphones.

From a mental health perspective, student support services may consider interventions that address underlying needs related to safety, belonging, self-esteem, and self-actualization. Potential strategies include dealing with phone-unavailable situations, enhancing face-to-face communication skills, providing strategies for coping with loneliness or negative self-perceptions, and supporting students in setting clear goals and finding meaning in their daily lives. Coping skills training should combine problem-focused coping for addressing underlying contributors to nomophobia with emotion-focused coping for regulating acute distress when students are unable to use smartphones.

Given the qualitative descriptive design of this study, these implications should be interpreted as context-informed recommendations rather than evidence of intervention effectiveness. Future longitudinal and intervention studies are needed to examine whether needs-based and coping-focused programs can reduce nomophobia.

### Research limitations

Our research presents some limitations. First, the students were second-year undergraduate nursing students from a single university. The relatively narrow sample may restrict the generalizability of the findings across different cultural or educational contexts. Second, most students in our study were female, and while female nursing students greatly outnumber males in nursing education (World Health Organization, 2020), this may limit the exploration of gendered experiences of nomophobia.

## Conclusion

This qualitative descriptive study explored the reasons for nomophobia and the coping strategies used by nursing students. The findings indicate that nomophobia is linked to the perceived disruption of smartphone-supported resources in daily functioning, learning, psychosocial support, and personal development. Interpreted through Maslow’s hierarchy of needs, smartphone unavailability may be understood as a perceived threat to students’ safety, belongingness, esteem, cognitive needs, and self-actualization. Besides, environmental conditions such as digital course design may contribute to nomophobia. In addition, students appear to use emotion-focused coping strategies predominantly, particularly interest-based attention-diverting activities, whereas their problem-focused coping strategies are limited. Future longitudinal, mixed-methods, and intervention studies are needed to further examine these findings and evaluate whether needs-based and coping-focused programs can reduce nomophobia among nursing students.

## Data Availability

The raw data supporting the conclusions of this article will be made available by the authors, without undue reservation.

## References

[ref1] Abdoli N. Sadeghi-Bahmani D. Salari N. Khodamoradi M. Farnia V. Jahangiri S. . (2023). Nomophobia (no mobile phone phobia) and psychological health issues among young adult students. Eur J Investig Health Psychol Educ 13, 1762–1775. doi: 10.3390/ejihpe13090128, 37754467 PMC10527744

[ref2] Aguilera-Manrique G. Márquez-Hernández V. V. Alcaraz-Córdoba T. Granados-Gámez G. Gutiérrez-Puertas V. Gutiérrez-Puertas L. (2018). The relationship between nomophobia and the distraction associated with smartphone use among nursing students in their clinical practicum. PLoS One 13:e0202953. doi: 10.1371/journal.pone.0202953, 30148870 PMC6110512

[ref3] Alsayed S. Bano N. Alnajjar H. (2020). Evaluating practice of smartphone use among university students in undergraduate nursing education. Health Prof. Educ. 6, 238–246. doi: 10.1016/j.hpe.2019.06.004, 38826717

[ref4] Anjana K. K. Suresh V. C. Sachin B. S. Poornima C. (2021). The relationship between nomophobia, mental health, and coping style in medical students. Indian J. Soc. Psychiatry 37, 207–211. doi: 10.4103/ijsp.ijsp_213_20, 42244313

[ref5] Bartlett M. L. Taylor H. Nelson J. D. (2016). Comparison of mental health characteristics and stress between baccalaureate nursing students and non-nursing students. J. Nurs. Educ. 55, 87–90. doi: 10.3928/01484834-20160114-05, 26814818

[ref6] Beauregard P. Arnaert A. Ponzoni N. (2017). Nursing students’ perceptions of using smartphones in the community practicum: a qualitative study. Nurse Educ. Today 53, 1–6. doi: 10.1016/j.nedt.2017.03.002, 28324823

[ref7] Bista B. Bhattrai B. Khadka N. (2017). Stress and coping mechanisms among nursing students in Kathmandu. J. Manmohan Mem. Inst. Health Sci. 3, 16–23. doi: 10.3126/jmmihs.v3i1.19175, 37609976

[ref8] Bragazzi N. L. Re T. S. Zerbetto R. (2019). The relationship between nomophobia and maladaptive coping styles in a sample of Italian young adults: insights and implications from a cross-sectional study. JMIR Mental Health 6:e13154. doi: 10.2196/13154, 30763254 PMC6505371

[ref9] Braun V. Clarke V. (2006). Using thematic analysis in psychology Qual. Res. Psychol. 3, 77–101. doi: 10.1191/1478088706qp063oa, 42347858

[ref10] Çelik İnce S. (2021). Relationship between nomophobia of nursing students and their obesity and self-esteem. Perspect. Psychiatr. Care 57, 753–760. doi: 10.1111/ppc.12610, 32853441

[ref11] Chen J. Yang Y. Shen L. Zhang X. Hu R. (2023). Nursing students’ expectations and career preferences before clinical placement in mainland China: a qualitative exploration. Nurse Educ. Pract. 67:103552. doi: 10.1016/j.nepr.2023.103552, 36669296

[ref12] Çobanoğlu A. Bahadir-Yilmaz E. Kiziltan B. (2021). The relationship between nursing students’ digital and smartphone addiction levels and nomophobia: a descriptive, correlational study. Perspect. Psychiatr. Care 57, 1727–1734. doi: 10.1111/ppc.12742, 33616201

[ref13] Daraj L. R. AlGhareeb M. Almutawa Y. M. Trabelsi K. Jahrami H. (2023). Systematic review and meta-analysis of the correlation coefficients between nomophobia and anxiety, smartphone addiction, and insomnia symptoms. Healthcare 11:2066. doi: 10.3390/healthcare11142066, 37510507 PMC10380081

[ref14] Edwards E. J. Taylor C. S. Vaughan R. S. (2022). Individual differences in self-esteem and social anxiety predict problem smartphone use in adolescents Sch. Psychol. Int. 43, 460–476. doi: 10.1177/01430343221111061

[ref15] Emmons R. A. (2003). “Personal goals, life meaning, and virtue: wellsprings of a positive life,” in Flourishing: Positive Psychology and the Life Well-Lived, eds. Keyes C. L. M. Haidt J. (Washington, DC: American Psychological Association), 105–128.

[ref16] Epstein I. Bertram M. (2019). Using students’ smartphones to learn a nursing skill: students’ perspectives. J. Nurs. Educ. Pract. 9:24. doi: 10.5430/jnep.v9n5p24

[ref17] Eskin Bacaksiz F. Tuna R. Alan H. (2022). Nomophobia, netlessphobia, and fear of missing out in nursing students: a cross-sectional study in distance education. Nurse Educ. Today 118:105523. doi: 10.1016/j.nedt.2022.105523, 36058115

[ref18] Essel H. B. Vlachopoulos D. Tachie-Menson A. (2021). The relationship between the nomophobic levels of higher education students in Ghana and academic achievement. PLoS One 16:e0252880. doi: 10.1371/journal.pone.0252880, 34133434 PMC8208529

[ref19] Farooq M. Rizvi M. A. Wajid W. A. Ashraf M. Farooq M. Javed H. . (2022). Prevalence of nomophobia and an analysis of its contributing factors in the undergraduate students of Pakistan. Cyberpsychol. Behav. Soc. Netw. 25, 147–153. doi: 10.1089/cyber.2021.0148, 35021897

[ref20] Fornés-Vives J. Garcia-Banda G. Frias-Navarro D. Rosales-Viladrich G. (2016). Coping, stress, and personality in Spanish nursing students: a longitudinal study. Nurse Educ. Today 36, 318–323. doi: 10.1016/j.nedt.2015.08.011, 26343997

[ref21] Gaber Hamzaa H. Atta M. H. R. Elghareap Hassan Elmetwally Omar M. Reda Fathy Abdel Majeed Machaly E. Mohamed Amin S. Mohamed Ibrahim Wahba N. (2024). Examining nursing students’ prevalence of nomophobia, and psychological alienation and their correlates with fear of missing out: a multisites survey. SAGE Open Nurs. 10:23779608241301223. doi: 10.1177/23779608241301223, 39691625 PMC11650578

[ref22] Gezgin D. M. Hamutoglu N. B. Sezen-Gultekin G. Ayas T. (2018). The relationship between nomophobia and loneliness among Turkish adolescents. Int. J. Res. Educ. Sci. 4, 358–374. doi: 10.21890/ijres.409265

[ref23] Gonçalves S. Dias P. Correia A.-P. (2020). Nomophobia and lifestyle: smartphone use and its relationship to psychopathologies. Comput. Hum. Behav. Rep. 2:100025. doi: 10.1016/j.chbr.2020.100025, 38826717

[ref24] Gözümi A. İ. C. Erkul R. Aksoy N. (2020). Use of smartphones in class: examining the relationship between M-learning readiness, cyberloafing, nomophobia and addiction variables. Int. J. Prog. Educ. 16, 94–120. doi: 10.29329/ijpe.2020.280.6

[ref25] Gutiérrez-Puertas L. Márquez-Hernández V. V. Gutiérrez-Puertas V. Granados-Gámez G. Aguilera-Manrique G. (2020). The effect of cell phones on attention and learning in nursing students. Comput. Inform. Nurs. 38, 408–414. doi: 10.1097/cin.0000000000000626, 32349025

[ref26] Hirsch C. D. Barlem E. L. D. Tomaschewski-Barlem J. G. Lunardi V. L. de Oliveira A. C. C. (2015). Predictors of stress and coping strategies adopted by nursing students. Acta Paul. Enferm. 28, 224–229. doi: 10.1590/1982-0194201500038

[ref27] Jahrami H. Fekih-Romdhane F. Saif Z. Bragazzi N. L. Pandi-Perumal S. R. BaHammam A. S. . (2022). A social media outage was associated with a surge in nomophobia, and the magnitude of change in nomophobia during the outage was associated with baseline insomnia. Clocks Sleep 4, 508–519. doi: 10.3390/clockssleep4040040, 36278533 PMC9589948

[ref28] Kang S. Jung J. (2014). Mobile communication for human needs: a comparison of smartphone use between the US and Korea. Comput. Hum. Behav. 35, 376–387. doi: 10.1016/j.chb.2014.03.024, 38826717

[ref29] King A. L. S. Valença A. M. Silva A. C. Sancassiani F. Machado S. Nardi A. E. (2014). “Nomophobia”: impact of cell phone use interfering with symptoms and emotions of individuals with panic disorder compared with a control group. Clini. Prac. Epidemiol. Mental Health 10, 28–35. doi: 10.2174/1745017901410010028, 24669231 PMC3962983

[ref30] Lazarus R. S. (1993). Coping theory and research: past, present, and future. Psychosom. Med. 55, 234–247. doi: 10.1097/00006842-199305000-00002, 8346332

[ref31] Lazarus R. S. Folkman S. (1984). Stress, Appraisal, and Coping. Berlin: Springer Publishing Company.

[ref32] Lincoln Y. S. Guba E. G. (1986). But is it rigorous? Trustworthiness and authenticity in naturalistic evaluation. New Dir. Program Eval. 30, 73–84. doi: 10.1002/ev.1427

[ref33] Lincoln Y. S. Guba E. G. (1988) Criteria for assessing naturalistic inquiries as reports. Proceedings of the Annual Meeting of the American Educational Research Association, Washington, DC: American Educational Research Association

[ref34] Liu Y. Zhang Q. Cui W. (2024). Latent profile analysis of nomophobia among nursing students and its relationship with a healthy lifestyle. Chin. J. Health Psychol. 33, 629–635.

[ref35] Maher A. J. (2025). Rigour in interpretive qualitative research in education: ideas to think with. Br. Educ. Res. J. 51, 2099–2115. doi: 10.1002/berj.4156, 41531421

[ref36] Márquez-Hernández V. V. Gutiérrez-Puertas L. Granados-Gámez G. Gutiérrez-Puertas V. Aguilera-Manrique G. (2020). Problematic mobile phone use, nomophobia and decision-making in nursing students mobile and decision-making in nursing students. Nurse Educ. Pract. 49:102910. doi: 10.1016/j.nepr.2020.102910, 33152615

[ref37] Maslow A. H. (1943). A theory of human motivation. Psychol. Rev. 50, 370–396. doi: 10.1037/h0054346, 27371692

[ref38] Maslow A. H. (1962). “Some basic propositions of a growth and self-actualization psychology,” in Perceiving, Behaving, Becoming: A New Focus for Education, ed. Combs A. W. (Washington, DC: American Psychological Association), 34–49.

[ref39] Maslow A. H. (1987). Motivation and Personality. 3rd Edn New York, NY: Harper & Row.

[ref40] Moreno-Guerrero A. J. Hinojo-Lucena F. J. Trujillo-Torres J. M. Rodríguez-García A. M. (2021). Nomophobia and the influence of time to REST among nursing students. A descriptive, correlational and predictive research. Nurse Educ. Pract. 52:103025. doi: 10.1016/j.nepr.2021.103025, 33865072

[ref41] Mudgal S. K. Sharma S. K. Gaur R. Sharma M. Latha T. Patidar V. (2024). Prevalence and severity of nomophobia among nurses: a systematic review and meta-analysis. Investigación y Educación en Enfermería. 42:e05. doi: 10.17533/udea.iee.v42n3e05

[ref42] Notara V. Vagka E. Gnardellis C. Lagiou A. (2021). The emerging phenomenon of nomophobia in young adults: a systematic review study. Addict. Health. 13, 120–136. doi: 10.22122/ahj.v13i2.309, 34703533 PMC8519611

[ref43] Nway N. C. Phetrasuwan S. Putdivarnichapong W. Vongsirimas N. (2023). Factors contributing to depressive symptoms among undergraduate nursing students: a cross-sectional study. Nurse Educ. Pract. 68:103587. doi: 10.1016/j.nepr.2023.103587, 36842294

[ref44] O’Connor S. Andrews T. (2018). Smartphones and mobile applications (apps) in clinical nursing education: a student perspective. Nurse Educ. Today 69, 172–178. doi: 10.1016/j.nedt.2018.07.013, 30096510

[ref45] Patzak A. Zhang X. (2025). Blending teacher autonomy support and provision of structure in the classroom for optimal motivation: a systematic review and meta-analysis. Educ. Psychol. Rev. 37:17. doi: 10.1007/s10648-025-09994-2, 30311153

[ref46] Pavithra M. Madhukumar S. Ts M. M. (2015). A study on nomophobia-mobile phone dependence, among students of a medical college in Bangalore. Nat. J. Commun. Med. 6, 340–344.

[ref47] Pavon J. U. (2020). Sense of Belonging in a Digital, Yet Social World: A Study on the Impacts of Social Media Use, Social Connectedness, and Psychological Well-Being Among College Students. Fresno, CA: California State University.

[ref48] Putri C. Wibowo D. H. (2024). Nomophobia and self-esteem: a study of migrant students’ psychological well-being. J. Bimbingan. Konsel. Terapan 8, 223–235. doi: 10.30598/jbkt.v8i2.2014

[ref49] Ramjan L. M. Salamonson Y. Batt S. Kong A. McGrath B. Richards G. . (2021). The negative impact of smartphone usage on nursing students: an integrative literature review. Nurse Educ. Today 102:104909. doi: 10.1016/j.nedt.2021.104909, 33894590

[ref50] Ranjan R. Balhara Y. P. S. Mishra B. R. Sarkar S. Bharti A. Sinha M. . (2024). Description of nomophobia among college students: an interpretative phenomenological analysis. Indian J. Psychol. Med. 46, 305–312. doi: 10.1177/02537176231219195, 39056034 PMC11268276

[ref51] Ren S. Gu L. Liu T. (2020). Revision of the Chinese version of nomophobia questionnaire. Psychol. Explor. 40, 247–253.

[ref52] Renninger K. A. Hidi S. (2015). The Power of Interest for Motivation and Engagement. London: Routledge.

[ref53] Rodríguez-García A.-M. Moreno-Guerrero A.-J. Lopez Belmonte J. (2020). Nomophobia: an individual’s growing fear of being without a smartphone—a systematic literature review. Int. J. Environ. Res. Public Health 17:580. doi: 10.3390/ijerph17020580, 31963208 PMC7013598

[ref54] Rogayan D. V. Jr. Abaniel J. C. A. (2025). Mobile technology, nomophobia, and student well-being: policy considerations for nursing education. Nurs. Outlook 73:102532. doi: 10.1016/j.outlook.2025.102532, 40835494

[ref55] Ryan R. M. Deci E. L. (2000). Self-determination theory and the facilitation of intrinsic motivation, social development, and well-being. Am. Psychol. 55, 68–78. doi: 10.1037/0003-066X.55.1.68, 11392867

[ref56] Safaria T. Rahayu Y. P. Rahaju S. (2025). “Oh my phone, I can’t live without you”: a phenomenological study of nomophobia among college students. Qual. Res. J. 25, 535–568. doi: 10.1108/qrj-06-2023-0092, 35579975

[ref57] Santl L. Brajkovic L. Kopilaš V. (2022). Relationship between nomophobia, various emotional difficulties, and distress factors among students. Eur J Investig Health Psychol Educ 12, 716–730. doi: 10.3390/ejihpe12070053, 35877453 PMC9316259

[ref58] Saunders S. Munro D. Bore M. (1998). Maslow’s hierarchy of needs and its relationship with psychological health and materialism. South Pac. J. Psychol. 10, 15–25. doi: 10.1017/S0257543400000833, 41292463

[ref59] Servidio R. (2023). Fear of missing out and self-esteem as mediators of the relationship between maximization and problematic smartphone use. Curr. Psychol. 42, 232–242. doi: 10.1007/s12144-020-01341-8, 30311153

[ref60] Turner A. (2025) How many smartphones are in the world? https://www.bankmycell.com/blog/how-many-phones-are-in-the-world (Accessed April 6, 2025)

[ref61] Uguz G. Bacaksiz F. E. (2022). Relationships between personality traits and nomophobia: research on nurses working in public hospitals. Perspect. Psychiatr. Care 58, 673–681. doi: 10.1111/ppc.12834, 33969498

[ref62] Vaismoradi M. Turunen H. Bondas T. (2013). Content analysis and thematic analysis: implications for conducting a qualitative descriptive study. Nurs. Health Sci. 15, 398–405. doi: 10.1111/nhs.12048, 23480423

[ref63] Wang Y. Ge Y. Chu M. Xu X. (2024). Factors influencing nursing undergraduates’ motivation for postgraduate entrance: a qualitative inquiry. BMC Nurs. 23:728. doi: 10.1186/s12912-024-02373-2, 39379915 PMC11462717

[ref64] Willis D. G. Sullivan-Bolyai S. Knafl K. Cohen M. Z. (2016). Distinguishing features and similarities between descriptive phenomenological and qualitative description research. West. J. Nurs. Res. 38, 1185–1204. doi: 10.1177/0193945916645499, 27106878

[ref65] World Health Organization (2020). State of the world’s nursing 2020: investing in education, jobs and leadership. Available online at: https://www.who.int/publications/i/item/9789240003279 (Accessed April 6, 2020)

[ref66] Yardley L. Bradbury K. Morrison L. (2021). Using Qualitative Research for Intervention Development and Evaluation. 2nd Edn Washington, DC: American Psychological Association.

[ref67] Yigit D. Cakirli M. Acikgoz A. (2024). The effect of nomophobia levels on nursing students’ depression, anxiety and stress levels. J. Eval. Clin. Pract. 30, 1490–1496. doi: 10.1111/jep.14071, 38943492

[ref68] Yildirim C. Correia A.-P. (2015). Exploring the dimensions of nomophobia: development and validation of a self-reported questionnaire. Comput. Hum. Behav. 49, 130–137. doi: 10.1016/j.chb.2015.02.059, 38826717

[ref69] Yousefian Z. Khodabakhshi-Koolaee A. (2023). The quality of social interactions in young girls with nomophobia syndrome. Comput. Human Behav. Rep. 12:100340. doi: 10.1016/j.chbr.2023.100340

[ref70] Zhang L. Wang B. Xu Q. Fu C. (2023). The role of boredom proneness and self-control in the association between anxiety and smartphone addiction among college students: a multiple mediation model. Front. Public Health 11:1201079. doi: 10.3389/fpubh.2023.1201079, 37564421 PMC10409989

[ref71] Zimmer-Gembeck M. J. (2021). Coping flexibility: variability, fit and associations with efficacy, emotion regulation, decentering and responses to stress. Stress. Health 37, 848–861. doi: 10.1002/smi.3043, 33720506

